# Ketogenic diet in clinical populations—a narrative review

**DOI:** 10.3389/fmed.2024.1432717

**Published:** 2024-10-29

**Authors:** Alon Zemer, Shabnam Samaei, Uri Yoel, Aya Biderman, Yair Pincu

**Affiliations:** ^1^Department of Pharmacology and Clinical Biochemistry, Ben-Gurion University of the Negev, Beer Sheva, Israel; ^2^Department of Health and Exercise Science, University of Oklahoma, Norman, OK, United States; ^3^Endocrinology Unit, Soroka University Medical Center, Beer Sheva, Israel; ^4^Department of Family Medicine, Goldman Medical School, Ben-Gurion University of the Negev and Clalit Health Services, Beer Sheva, Israel; ^5^Harold Hamm Diabetes Center, Oklahoma City, OK, United States

**Keywords:** very-low-carbohydrate-diet, obesity, diabetes, weight-loss, cancer, intractable pediatric epilepsy, neurodegenerative disease

## Abstract

Ketogenic diet (KD) is a high-fat, low-carbohydrate (CHO) diet, designed to induce a metabolic state of ketosis in which the body metabolizes primarily lipids for energy production. Various forms of KD are being promoted as promising treatments for numerous health conditions from chronic headaches to weight-loss and even different forms of cancer and are becoming increasingly more popular. KD appears to be an efficacious approach for weight-loss, and maintenance, improved glycemia, cognitive function and cancer prognosis. However, there is a controversy regarding the safety of KD, and the potential health risks that might be associated with long-term exposure to KD. There is a gap between the acceptance and utilization of KD in individuals with health conditions and the criticism and negative attitudes toward KD by some clinicians. Many individuals choose to follow KD and are encouraged by the positive results they experience. Although the medical establishment does not endorse KD as a first line of treatment, clinicians need to be informed about KD, and offer support and medical supervision for patients who self-select to follow KD. This can ensure that within the boundaries of KD, patients will make good and healthy dietary choices and prevent clinical disengagement in extreme cases. To that end, there is an urgent need for good quality research to address the issues of long-term safety of KD in different clinical populations and for standardization of KD both in research and in the clinic.

## 1 Introduction

Ketogenic diet (KD) is a high-fat, low-carbohydrate (CHO) diet (1), in which the amount of daily CHO consumption is < 50 or even < 20 g/day—approximately 5–10% of total daily caloric intake (2–4). This is equivalent to 1–3 CHO exchange servings (e.g., 1–3 slices of bread) a day. Under fasting conditions or CHO restriction, metabolic products of lipid β-oxidation in the liver form ketone bodies [including acetoacetate and β-hydroxybutyrate (β-OHB)], which are also oxidized to produce energy. The accumulation of ketone bodies in the circulation is termed ketosis (serum β-OHB = 0.5–5 mmol/l) and represents a physiological response to low CHO availability. Various forms of KD are being promoted as promising treatments for numerous health conditions like chronic headaches (5), psychiatric diseases (6), hypertension (7), autoimmune diseases (8, 9), polycystic syndromes (10, 11), and even different forms of cancer (12). Many individuals utilize a KD for weight-loss, due to its contribution to increased appetite control, and reduced adiposity (13), and KDs are increasingly becoming more popular (14).

Nevertheless, there is a profound controversy regarding the safety of KD, and the potential health risks that might be associated with long-term exposure to KD. For example, one concern is the increased risk for cardiovascular disease (CVD) from chronically elevated levels of circulating lipids and lipoproteins—particularly LDL (15). However, conflicting evidence show both increases and decreases in LDL cholesterol following KD and several research findings suggest that KD may in fact be protective against CVD (see Potential Risks below). One of the contributing factors to the conflicting research evidence is the lack of standardization in the application of KD and the verification of metabolic ketosis in study participants. Some studies do not provide any criteria for KD while others use net carbohydrate content or percent of total caloric intake to classify KD and similarly, some studies do not evaluate metabolic ketosis while others measure ketone in the blood or urine or measure the ratio between albumin or urea to creatinine. Please refer to Table 1 for a summary of carbohydrate restriction criteria and ketosis assessment in all the references with human participants used in this review.

**Table 1:** Ketogenic diet characteristics in human studies.

	**No. of publications**	**Reference no**.
**Carbohydrate restriction criteria**		
No specific criteria provided	6	(25, 45, 113, 117, 135–139)
75 < CHO < 130 g/d or 10–35% of total kcal	5	(54, 59, 125, 130, 138)
20 < CHO < 75 g/d or 5% < CHO < 10% of total kcal	20	(32, 36–38, 40, 47, 51, 53, 57, 58, 60, 61, 81, 87, 104, 119, 124, 132, 140, 143)
CHO < 20 g/d or < 5% of total kcal	20	(22, 33, 39, 43, 49, 50, 83, 108, 109, 111, 116, 118, 121, 122, 129, 131, 136, 137, 142, 144)
Fat: CHO ratio (2:1 to 8:1) or low-carb diet score^*^	6	(26, 27, 86, 112, 114^*^, 115^*^)
**Ketosis assessment**		
Blood ketones	22	(27, 32, 36, 47, 53, 57, 60, 61, 81, 87, 104, 108, 109, 122, 129, 132, 135–138, 140, 142)
Urine ketones or albumin/urea to creatinine	19	(22, 32, 33, 37, 39, 45, 49–51, 57, 111, 129, 132, 135–137, 140, 142, 143)
No ketosis measurement	21	(26, 38, 40, 43, 54, 57–59, 113–119, 121, 124, 130, 131, 139, 144)

CHO, carbohydrates; kcal, kilocalories. ^*^Studies that used a low-carb diet index as the CHO restriction criteria.

This review is written from a clinician perspective and is aimed to introduce KD to primary care practitioners and to summarize the benefits and challenges of KD. This is not meant to be a comprehensive review, and an in-depth description of the physiology of KD is beyond the scope of this paper. In this review, we will explore some of the different ways in which KD is being used in the treatment of several medical conditions, while addressing some potential risks of KD in clinical populations. We focus on pediatric epilepsy, weight-loss, diabetes management, neurodegenerative diseases and cancer.

## 2 Pediatric epilepsy

Epilepsy is characterized by recurrent seizures—brief episodes of involuntary movements, caused by excessive electrical activation in different neurons of the brain. Current prevalence is estimated to be 760 per 100,000 people worldwide, making it one of the most common neurological diseases globally (16). Although different dietary interventions were used to treat seizures since 500 BC, KD was introduced in the early 1920′s, as a novel way to treat pediatric epilepsy (17). Some mechanisms were suggested for the beneficial effects on epileptic seizures, from synaptic protection through activation of K^ATP^ channels (18), to anti-inflammatory (19), and antioxidant effects that were associated with ketosis (20, 21). The introduction of modern anti-epileptic drugs in the late 1930′s, resulted in near complete abandonment of KD as treatment for epilepsy. Moreover, the difficulty in meeting the strict dietary limitations associated with KD and as a result failing to maintain a proper state of ketosis made this treatment regime not sustainable for many, and also played a role in its neglect (17). Even though later work demonstrated that less drastic low-CHO diets also presented beneficial seizure-moderating effects (22), anticonvulsant drugs eventually replaced most of the dietary interventions for epilepsy (17).

Nowadays an estimated 20–30% of epileptic patients experience drug-resistance and do not benefit from the available antiepileptic medications (23, 24). For such patients, KD is still a viable and successful treatment option (25). The effects of KD on epilepsy appear to be age dependent, as several studies in drug-resistant pediatric epilepsy report higher rates of seizure cessation in infants compared to older kids. Although KD shows great promise, resulting in complete elimination of seizures in approximately one third of infants who were < 1.5 years old at initiation of KD, these rates were much lower in children who were >1.5 years old at initiation of KD (26). Similar results were reported when KD was compared to the less strict modified Atkins diet (MAD). KD was more favorable in seizure reduction in infants with refractory epilepsy under 2 years of age, these differences were not replicated in children 2–6 years and 6–18 years old. The proportion of subjects with >50% reduction in seizure prevalence at 6 months was 59, 38, and 22% vs. 45, 41, and 19% in KD vs. MAD in infants < 2, 2–6, and 6–18 years old, respectively (27). Other studies also report that the rates of seizure reduction in children with epilepsy on KD were not fully replicated in adults, and that the effects of KD intervention in epileptic adults are less conclusive (25).

Although some potential adverse effects might exist in long-term KD in young children, like bone fractures, kidney stones and delayed growth, these were reported in only one study and the research evidence is not strong (2). KD is still an efficacious treatment option, recommended by physicians in cases of medication-resistant epilepsy (28). Current (2018) guidelines of the International Ketogenic Diet Study Group, recommend to strongly consider KD in children who failed 2 antiseizure drugs, and that the specific KD used should be individualized for the child and their family—while being supported and guided by a KD team, including a neurologist and a nutritionist (2). The KD team should see the children every 3 months in the first year of KD initiation and every 6 months thereafter. In addition, it is recommended that children on a KD will be supplemented with multivitamins and calcium to prevent potential deficiencies. The group also states that discontinuation of KD is recommended after 3 months if not efficacious in reducing seizures, but if seizure control is nearly complete (>90% reduction), and no side effects are apparent, KD can be practiced for several years. In most cases KD is terminated after 2 years of initiation (2). Notably, very similar recommendations were made more recently (2023) by the Italian League Against Epilepsy Dietary Therapy Study Group (29).

## 3 Weight-loss

Obesity is a state of excess adipose tissue that adversely affects health. Clinically, obesity is determined as a body mass index (BMI) ≥ 30 kg/m^2^. The prevalence of adult obesity in the USA is 41.9% while 73.6% of Americans are overweight (BMI > 25), and rates are at a constant rise also in children (30).

The etiology of obesity is multifactorial, but excessive CHO consumption—particularly highly processed CHO is a major cause for obesity and its comorbidities (31). CHO comprises ~55% of the macronutrients in the Standard American Diet (SAD) (14). KD is very effective in inducing weight-loss, as early as 2 weeks (32) and even when compared to some weight-loss medications like Orlistat (33). This is attributed at least in part, to the low-CHO content which increases lipolysis and lipid oxidation and reduces adipose tissue mass (34). Many individuals living with obesity struggle with weight-loss and the promise of KD makes it very popular for weight-loss—even in individuals who are not clinically categorized as obese (35).

Several measures of adiposity were improved following 10 weeks of KD, compared to SAD in overweight individuals (36). Similarly, several studies report that 6 months of KD resulted in a significant weight-loss, in a quasi-experimental design (37) as well as in randomized controlled trials, when compared to a low-calorie, low-fat diet (38, 39). Moreover, the weight-loss and improved body composition are also associated with reduced blood glucose and lipids (38). Additionally, compared to low-fat control diet, KD also resulted in increased resting energy expenditure and thermic effect of feeding following weight-loss, which also makes KD a good strategy for weight maintenance (40).

These findings suggest that KD should be recommended as a potent and safe strategy for weight-loss and improved metabolism in individuals living with obesity. However, some publications suggest that these beneficial effects might not persist in the long term (>24 months). For example, a meta-analysis of 13 articles analyzing the effect of 12 months of KD in obesity concluded that although KD is a beneficial tool for weight-loss, the rate of weight-loss was lower during the 7–12 months compared to the first 6 months of the diet (41). Moreover, KD may result in a greater loss of muscle mass compared to low-fat diets (42). A comprehensive study testing changes in metabolic rate (RMR) and body composition found that 4 weeks of KD resulted in weight-loss and a slight increase in RMR, but lean mass declines were greater compared to high-CHO diet (32). Conversely, 52 weeks of calorie-restricted KD did not impair muscle strength or physical function and had similar changes to body composition compared to isocaloric high-fat diet (43). The deleterious effects of KD on muscle mass are yet to be corroborated, but the proposed mechanisms might be a combination of lower insulin and higher corticosterone in the blood, and decreased secretion of insulin-like growth factor (IGF-1) and inhibition of muscle protein synthesis (42). Maintaining adequate protein content in the KD might help to preserve muscle mass during weight-loss, but more research is needed to fully appreciate the effects of KD on muscle mass and protein synthetic activity, especially when physical activity is incorporated as a complimentary weight-loss strategy (42).

In summary, KD is a very efficacious approach for weight-loss, and maintenance, with some reported adverse effects, however with no strong evidence to determine that these adverse effects, if indeed truly exist, outweigh the potential benefits of KD. Additionally, although long-term use of KD (>1 year) for weight-loss was successful, it did not result in superior weight-loss rates compared to other dietary approaches.

## 4 Diabetes management

Diabetes affects millions of people worldwide and causes various health complications including heart disease, blindness, renal failure, and peripheral nerve damage. Type 1 diabetes (T1D) is characterized by a complete insulin deficiency and required exogenous insulin administration. Type 2 Diabetes (T2D) is typically characterized by insulin resistance, accompanied by hyperinsulinemia and hyperlipidemia and is highly associated with adiposity. There is no cure for diabetes (T1D or T2D), and current clinical goals are successful diabetes management using diet, physical activity and medications, aimed at controlling glycemia (HbA1C < 7%) (44). KD seems to be beneficial for controlling blood glucose primarily due to the restricted CHO and reliance on ketones for energy production. KD has also been found to decrease circulating markers of systemic inflammation, which can contribute to the improved glycemia (45). In fact, CHO restriction as a form of diabetes treatment is not a new concept and was the only available treatment in the pre-insulin era. In his textbook from 1892, William Osler describes a dietary treatment for diabetic patients, prescribing a diet with only 3% CHO but high in fat (65%)—very similar to modern day KD (46). The benefits of KD for individuals with T2D also include increased lipolysis, significant weight-loss and reduction of blood triglycerides (TG), and together with other changes these result in decreased insulin resistance (47). However, there is a scarcity of evidence as to the long-term efficacy of KD in maintaining improved glycemia in diabetes or whether the potential risks associated with KD might outweigh its potential metabolic benefits (48).

### 4.1 Type 2 diabetes

Several short-term studies (< 24 weeks) in individuals with T2D consistently show significant weight-loss (6–11% decrease), reduced HbA1C (16–17% reduction) and high rates of cessation of diabetes medications (80–95% of participants) following KD (49, 50), even if KD was administered as an online intervention (51); This is quite astonishing. However, there is limited research evidence that these beneficial outcomes persist in long-term KD treatment. For example, a meta-analysis of 36 trials (*n* = 2,161) comparing KD to low-fat diet reported that while HbA1C levels decreased more with KD in the short-term (< 12 months), by 1 year the mean difference between the diets was reduced by ~75% (52). Although other studies report significant weight-loss and improved glycemic control (decreased HbA1c and cessation of diabetes medications) following 12 months of KD (53), in their meta-analysis van Zuuren et al. report that difference in these beneficial effects between the diet groups completely disappeared at 2 years (52). One of the suggested reasons for the diminished effects of KD with time is the difficulty in long-term adherence to KD (52, 54), however adherence was not evaluated in this analysis (52).

An interesting alternative to KD, that is based on the metabolic principles of CHO-restriction was suggested by Chang et al. (55), using a very-low-CHO high fat breakfast with moderate CHO consumption during the rest of the day. The authors reported decreases in overall postprandial glycemia, glycemic variability and over-all hunger sensation following this ketogenic breakfast approach in 23 adults with T2D. This strategy might be sufficient to promote the metabolic benefits of KD in individuals with T2D but without the potential long-term risks of KD—but more research is required before this can be determined.

### 4.2 Type 1 diabetes

The use of KD in T1D as a complimentary strategy to insulin treatment is gaining popularity since optimal glycemic control (HbA1C < 7%) is only achieved by 15.8% of individuals with T1D and that dietary CHO has been identified as a major cause for glycemic excursions (56). Conversely, an observational study, albeit smaller in scale (*n* = 11 patients with T1D), on self-selected long-term KD (2.6 ± 3.3 years), showed remarkable HBA1c levels (5.3 ± 0.4%) and long duration of euglycemic time (74 ± 20% of the time). Nevertheless, 0.9 daily episodes of hypoglycemia were noted (3.6% of the time with blood glucose < 3.0 mmol/l), and dyslipidemia was prominent, as total cholesterol, LDL, and total-cholesterol/HDL-cholesterol ratio were above the recommended range in over 60% of participants. Notably, HDL levels remained within the recommended range (57). Similarly, an online survey of over 300 adults and children with T1D who followed KD for ~2 years on average, reported a mean HbA1C = 5.67 ± 0.66% with negligible number of adverse events (58). This again, is astonishing, considering the large sample size and that this HbA1C value is similar to that of individuals with no diabetes.

The benefits of restricting CHO in individuals with T1D are apparent in several other studies, showing stable 24-h glycemia, a blunted postprandial glucose response and lower insulin dosing (59, 60). A study using continuous glucose monitors in individuals with T1D, treated with an insulin pump reported that only 1 week of KD resulted in increased time in glucose target range (TIR) and decreased time below target range (TBR) and decreased glucose variability (61). However, a more moderate restriction of CHO consumption might be enough to achieve beneficial metabolic outcomes in individuals with T1D. Krebs et al. (62), utilized a moderate CHO-restriction strategy, with CHO consumption restricted only to < 75 g/day and not to the typical < 50 g/day commonly used in KD. This resulted in improved glycemic control and reduced insulin requirements without a significant weight-loss following 12 weeks on that diet. Additionally, no significant changes in blood glucose variability or blood lipid profile were found (62). The long-term effects of such approach, and how it compares to low-fat diet or KD remains to be examined.

When reviewing current literature, even with the increasing reports of beneficial outcomes of KD in individuals with T1D, there is still a considerable lack of adequate evidence, particularly long-term randomized controlled trials, to support a generalized clinical recommendation to use KD in individuals with T1D. In a recent review by Dr. David Ludwig—a longtime advocate for KD, several very compelling arguments were made in favor of using KD in T1D, primarily addressing the contribution of CHO load to glycemic excursions and the potential for KD to improve glycemia, minimize daily insulin requirements, and reduce glucose variability. Interestingly, the authors also describe some of the issues with current evidence and mention small sample size and selection bias, short term interventions, and flaws in dietary assessment in the studies they analyzed in that review (63).

Currently, the popularity of KD in individuals with T1D is increasing, particularly among highly educated individuals, in a middle to upper socioeconomic status. These individuals are highly motivated and involved in their medical care and have a good understanding of diabetes physiology and technology (56). Current clinical recommendations do not advocate for KD (64) and in the case of children and adolescents with T1D even recommend against it (65). This can lead to conflicts between the patients and the clinical team, which might be perceived by the patients as dismissive and condescending and this might lead to miscommunication and even clinical disengagement of the patients (56).

We believe that the metabolic benefits of KD and its efficacy in weight-loss and improving glycemia, particularly as they relate to individuals with diabetes cannot be ignored. Encouraged by the positive effects on glycemia and body weight, more patients adopt KD as a dietary approach. This means that clinicians cannot and should not dismiss KD as a dietary approach to treat diabetes. However, since some of the published work has different methodological issues that affect the robustness of the current evidence, they highlight the urgent and profound need for well-designed, adequately powered, long-term randomized controlled trials to assess its safety, potential risks, and health outcomes when applied as a clinically mandated and clinically supported treatment. Such research might help to make KD more acceptable in the medical establishment as an efficacious dietary approach for individuals living with obesity and diabetes.

## 5 Cancer

Cancer is the second leading cause of death in the United States, responsible for 22% of annual fatalities (66). Carcinogenesis results from mutations disrupting genes associated with cellular growth processes (67, 68), often caused by exposure to environmental mutagens combined with deficiencies in DNA repair mechanisms (69). Most tumor cells display a shift to aerobic glycolysis as their main energy pathway (70). Since ketone metabolism inhibits glycolysis, the prime bioenergetic pathway for many tumors (71, 72), cancer cells are vulnerable to CHO-restriction approaches such as KD (73).

In recent years, much focus has been directed to recognizing obesity as a major risk factor for many cancer types (74), prompting increased focus on lifestyle approaches. The potent weight-loss effect of KD makes it an attractive approach to reduce cancer risk by decreasing obesity. Consequently, several clinical investigations conclude that appropriate nutrition can significantly improve outcomes as a complementary therapy (75).

One proposed mechanism linking obesity and cancer involves elevated circulating insulin and insulin-like growth factor 1 (IGF-1) levels, which over-activate the IGF system (76). This may promote tumor proliferation and progression by interfering with mitogenic signaling pathways (12, 77–80). KD reduces blood glucose and lowers insulin and IGF-1 activity (81) and provides a metabolic strategy to potentially slow cancer cell expansion. KD also attenuate peritumoral inflammation and edema, key factors enabling invasion and metastasis (9). Hence, KD shows promising translational potential for integrated cancer treatment (82, 83).

It appears that KD may have a beneficial impact particularly in tumors of the central nervous system. Evidently, the most substantial evidence for KD suppressing tumor growth and progression comes from studies on glioblastoma (84). This is achieved by counteracting their metabolism, reducing inflammation, modulating pathological gene transcription, and influencing the tumor microenvironment (85).

Evidently, a recent meta-analysis showed that KD resulted in a significant reduction in tumor weight and volume as well as prolonged survival time in rodents (86). Similar results were reported in humans, showing a significant reduction in tumor growth and size (87–89), and improved survival (90).

In the longest study to date, that examined the clinical efficacy of KD in women with advanced breast cancer over 10-year, 12 months of KD resulted in markedly improved outcomes compared to those who did not use KD (83). However, a study in rats found that long-term KD led to enhanced growth of renal tumors, attributed to increased growth hormone and fluctuations in IGF-1 levels (91). This highlights the need for further research into the long-term impacts of KD on cancer development and progression across different organ systems.

While KDs show promise as an adjuvant cancer therapy, they may cause adverse effects in some patients. Multiple studies have reported fatigue, muscle cramps, hypotension, constipation, and unintentional weight-loss in patients on KD (5, 85). Current evidence suggests KD may work best in combination with conventional or targeted treatments, and may have the greatest benefit when initiated early in cancer progression (92). KD may improve outcomes and quality of life for cancer patients, but more research is needed to test KD efficacy in treating a wide variety of tumors, evaluate KD risk in cancer patients and define clinical guideline for KD use in cancer treatment for maximum therapeutic gain.

## 6 Neurodegenerative diseases

Neurodegeneration is a common characteristic of numerous debilitating, incurable diseases, and the prevalence of these conditions is increasing rapidly (93). A broad range of neurodegenerative disorders impacts the central nervous system (CNS), disrupting sensory, motor, and cognitive functions such as vision, hearing, movement, speech and language, and memory (94). Here, we will focus on the two most common neurodegenerative diseases -Dementia and Parkinson's Disease.

### 6.1 Dementia

Dementia is a general term for a decline in cognitive ability, severe enough to interfere with daily function. Alzheimer's disease (AD) is the most common type of dementia, accounting for at least two-thirds of dementia cases in people 65 and older (95). Despite extensive efforts toward prevention and remediation, dementia remains an urgent public health issue, affecting over 50 million people worldwide (96). The pathogenesis of AD involves brain anatomic changes, including impaired neuronal glucose metabolism and accumulations of amyloid-β plaques and neurofibrillary tangles (97). Impaired glucose metabolism and insulin resistance have been strongly associated with progressive cognitive deficiency, therefore, the beneficial effects of KD on glycemia and insulin resistance might be protective against AD progression (98–100).

Several studies in animal models of AD showed a positive effect of KD on age-related cognitive decline (101, 102). In humans, a pilot study in older adults at risk for AD showed that a modified Mediterranean-KD was well-tolerated and was associated with improved outcomes of AD compared to the American-Heart-Association diet (103). Moreover, KD also resulted in improved daily function and quality of life in older adults with AD (104, 105).

### 6.2 Parkinson's disease

Parkinson's disease (PD) is another prevalent neurodegenerative disorder, characterized by gradual degeneration of dopamine-producing neurons. Over 1% of people over 60 are impacted by PD. The neuronal damage leads to progressive motor dysfunction and cognitive difficulties (106, 107). People with PD have impaired neuronal glucose metabolism so reliance on ketone metabolism during KD may normalize neuronal metabolism and slow the deterioration in dopamine synthesis (106, 108). Evidence that KD significantly improved motor functions in PD exist from both animal models (107) and humans (108). Additionally, a significant improvement in Parkinson Anxiety Scale scores was also reported (109). KD in patients with PD was found to be both feasible and safe, and resulted in notable improvements in both motor symptoms (tremors, rigidity, balance) and non-motor symptoms (sleep, mood, cognition) (107–109).

Taken together, these studies suggest KD may be a practical complementary therapy that can enhance functional abilities and quality of life for those living with PD, possibly by optimizing neuronal energy metabolism or other protective mechanisms.

## 7 Potential risks

KD is not suitable for everyone, and although data regarding long-term complication is limited, some non-life-threating adverse effects, such as constipation or diarrhea, headaches, halitosis, muscle cramps, general weakness and micronutrients deficiencies were mentioned (110–112). Several studies reported increased all-cause mortality risk in individuals on a KD diet (113–115), but in the same cohort, mortality risk was comparable in individuals on a high-CHO diet (114). Interestingly, vegetable-based KD was associated with lower all-cause mortality risk compared to both high-CHO diet and animal-based KD (114, 115). One of the major concerns of KD, attributable to the high animal fat consumption, is its effect on the lipid profile and a possible increase of CVD risk over time. Evidently, Goldberg et al., reported a case series in which five patients on KD demonstrated extreme hypercholesterolemia (three of five patients with cholesterol >500 mg/dl) (116). Similarly, another case series reported three patients with a marked acute elevation of total and LDL-cholesterol in otherwise healthy patients on KD (117). Interestingly, cholesterol levels were lowered in all patients who stopped KD (116, 117) or remained on KD but initiated lipid-lowering medication treatment (116). Similar evidence of KD resulting in hyperlipidemia exist from randomized controlled studies (118, 119), and meta-analyses (41, 120), but other studies found minimal change in lipids (121) or even improved lipid profiles following KD in overweight and obese individuals (37–39).

A comprehensive study that examined the effects of KD on markers of CVD in individuals with T2D concluded that 2 years of KD has beneficial effects on lipidemia and did not increase CVD risk (122). The authors found that in addition to attenuation of atherosclerotic progression (measured as central intima-media thickness), there was a decrease in small LDL particles (which are associated with diabetic dyslipidemia and increased CVD risk), and an increase in the less deleterious larger LDL particles (which resulted in the increase in total LDL cholesterol) (122). Interestingly, meta-analyses summarizing intervention studies ranging from 6 to 24 months, report elevated LDL following KD, but an increase in HDL was also reported in these studies (41, 120, 123), which might offset the CVD risk.

The concept of Lean Mass Hyper-Responder (LMHR) was introduced in 2017, describing people who are consuming KD, with lean BMI (< 25 kg/m^2^), very high LDL (≥200 mg/dl) along with elevated HDL (≥80 mg/dl), and lower TG ( ≤ 70 mg/dl) (124). This phenotype of elevated LDL cholesterol in the absence of other atherosclerotic CVD risk factors (such as obesity, low HDL, high TG) was verified in several publications (124, 125) and might even be relevant in individuals with type 1 diabetes [see Table 1 in (57)]. LMHR phenotype has recently been shown not to be associated with increased coronary artery plaque (126). Budoff et al., compared markers of coronary plaque (Coronary artery calcium and coronary computed tomography angiography) between a sample of 80 individuals consuming KD for 4.7 ± 2.8 years and a matched cohort from the Miami Heart Study [MiHeart, a prospective study of subclinical CVD risk factors in asymptomatic adults (127)]. Budoff et al., report that while the participants on KD had elevated mean LDL (272 ± 91 vs. 123± 38 mg/dl in the KD vs. MiHeart, respectively), they also had elevated HDL (90 ± 20 mg/dl), and lower TG (64 ± 23 mg/dl), compared to HDL 63 ± 19 mg/dl and TG 96 ± 45 mg/dl in the MiHeart cohort. Despite this observation, measures of coronary plaque were not different between the groups which suggests that while on KD, high levels of LDL together with elevated HDL and low TG do not result in increased atherosclerotic CVD risk (126). Although Budoff et al., provide quite convincing evidence to support this conclusion, they also acknowledge some limitations of their work including relatively low sample size, a cross sectional design and not profiling LDL particle size distribution (126). Additionally, they (126), and others (128) warrant caution, recommend personalized CVD risk assessment and call for more research to test the effects of increased LDL in the absence of other CVD risk factors on atherosclerotic CVD progression.

Using flow mediated dilation (FMD, a measure of endothelial function) to evaluate CVD risk yielded conflicting results. Compared to high-CHO diet, 8 weeks of KD did not impair FMD and also resulted in improvements in several blood markers of CVD risk (129). Similarly, while in a 2016 study of 115 patients living with obesity and T2D, after consuming an energy-restricted-high-carbohydrate (53% of energy) or isocaloric KD (14% carbohydrate, < 50 g/day) for 52 weeks (~13 months), Wycherley et al. found no difference in FMD between diet groups (130); In an earlier study (2010), they reported that both diets resulted in improvements in pulse wave velocity (a measure of arterial function), and circulating markers of endothelial function but the KD resulted in impaired FMD (131). Notably, in both studies, the authors reported similar glycemic improvements and weight-loss between the two isocaloric diet groups (130, 131). Interestingly, a similar study using the same design (52 weeks of energy restricted isocaloric diets) reported that although the energy-restricted KD resulted in similar weight-loss and glycemic control as the isocaloric high-carbohydrate diet, it resulted in improved lipid profile (132).

Together, these findings suggest that in most individuals, KD is not a major CVD risk factor while in others, which we currently cannot predict, KD might be associated with increased CVD risk. It is important to acknowledge that by inducing significant weight-loss, KD might in fact reduce CVD risk, since obesity is recognized as a major CVD risk factor (133). Changes in lipidemia are common in KD and are dependent upon BMI and metabolic health. While in individuals with obesity or type 2 diabetes KD does not invoke hypercholesterolemia (125, 134), increased LDL and total cholesterol are more common in lean individuals on KD. However, since a concurrent decrease in the TG/HDL ratio is also observed, this increase is not associated with elevated atherosclerotic CVD risk, as evaluated in individuals following approximately 5 years on KD (126). Other possible reasons for the discrepancy in lipidemic responses and the degree of vascular function in response to KD might be related to study design and study duration, however, important factors related to diet composition and total caloric content might also play an important role. These include the total amount of CHO and the degree of ketosis, the source and quality of dietary fats (e.g., margarine vs. bacon vs. olive oil and salmon) and the inclusion of fruit and vegetables. In most studies—these factors are not evaluated or not reported (see Table 1). It seems that genetic predisposition for hyperlipidemia might play a lesser role (116), but it is yet to be systematically evaluated in a long-term study. Since its growing popularity and widespread use, particularly for weight-loss, it is imperative to understand the long-term effects of a KD on blood lipids and consequently its effects on CVD risk.

In addition to the CVD risk concern, when considering KD in individuals with T1D, some believe a potential concern is diabetic ketoacidosis (DKA), an acute metabolic complication which result in excessive ketone accumulation and acidosis (63, 135). A few case reports of DKA in the background of KD were published (136–138), but these reports include starvation (136), undiagnosed T1D (137), or misdiagnosed diabetes (138), which were likely the cause for the DKA, rather than KD. Moreover, no well-designed randomized controlled studies were found to suggest this is a widespread phenomenon and it seems that KD is not associated with DKA in individuals with T1D. Evidently, when measuring circulating β-OHB in a group of individuals with T1D, on a voluntary KD, Ozoran et al., found mean (±SD) β-OHB levels considerably lower (1.2 ± 0.14 mmol/l) than the DKA threshold of >5 mmol/l (139). Notably, several reports describe the increased risk for euglycemic DKA in individuals with diabetes treated with sodium-glucose cotransporter-2 (SGLT-2) inhibitors and KD (140, 141). Concurrent SGLT-2 treatment and KD should be avoided.

Another concern of KD in individuals with T1D is related to the high intake of protein, which may increase patients' risk for renal dysfunction (142). However, 12 months of KD did not have any adverse effects on renal function in individuals living with obesity (143) or T2D (144). KD was also well-tolerated and safe, with no change in renal outcomes in a group of individuals living with T2D with mild diabetic kidney disease, albeit the intervention was very short (~4 months) (145).

Based on the current evidence it seems that for most individuals with T1D, KD do not hold significant risks that outweigh the potential benefits, and it is important to acknowledge that the magnitude of benefits of KD as an adjunctive therapy in T1D cannot be overlooked. Moreover, the significant effects of KD on weight-loss and glycemia, and the relative low risk make KD an attractive therapeutic strategy, at least for periods of under 1 year. It is important to consider however, that a more moderate CHO restriction (< 75 g/day) also resulted in improved measures of glycemic control (62) and might be more appropriate for patients who cannot follow KD for various reasons.

## 8 Conclusions

KD seems to hold several benefits that can improve outcome in various clinical populations. It is becoming more and more popular but is still not endorsed by the medical establishment. In some cases, there is a gap between the widespread acceptance of KD and its utilization in individuals with health conditions and the criticism and negative attitudes toward KD by some clinicians. The metabolic benefits of KD, its efficacy in inducing weight-loss and its beneficial effects on some neurodegenerative diseases cannot be overlooked. KD appears to be well-tolerated and safe in most cases. However, there is a serious lack of well-designed, properly powered randomized controlled trials that explore KD long-term efficacy and safety—particularly the consequences of elevated LDL in the absence of other CVD risk factors.

With the exception of intractable pediatric epilepsy, KD is currently not recommended by the different clinical societies as an acceptable treatment for conditions like obesity, diabetes, cancer, and neurodegenerative diseases. However, many individuals choose to follow KD and are encouraged by the positive results they experience. We, as clinicians, need to find ways to contain our patients' choices, educate ourselves about KD, and offer our support and our medical supervision. This can ensure that within the boundaries of KD, our patients will make good and healthy dietary choices and include low-carb vegetables and healthy dietary fats and avoid highly processed products that are marketed as “Keto-friendly”. This is important both to ensure healthy choices and to prevent clinical disengagement in extreme cases where conflicting attitudes toward KD and miscommunication might lead to a breach of trust between the patient and the clinician.

There is an urgent need for good quality research to address the issues of long-term safety of KD in different clinical population and for standardization of KD both in research and in clinical applications. Based on such research, recommendations should be made to include or to avoid KD as a therapeutic strategy for different individuals, in different health conditions.
